# Identification of Highly Selective and Potent Histone Deacetylase 3 Inhibitors Using Click Chemistry-Based Combinatorial Fragment Assembly

**DOI:** 10.1371/journal.pone.0068669

**Published:** 2013-07-16

**Authors:** Takayoshi Suzuki, Yuki Kasuya, Yukihiro Itoh, Yosuke Ota, Peng Zhan, Kaori Asamitsu, Hidehiko Nakagawa, Takashi Okamoto, Naoki Miyata

**Affiliations:** 1 Graduate School of Medical Science, Kyoto Prefectural University of Medicine, Kyoto, Japan; 2 PRESTO, Japan Science and Technology Agency (JST), Saitama, Japan; 3 Graduate School of Pharmaceutical Sciences, Nagoya City University, Nagoya, Japan; 4 Graduate School of Medical Sciences, Nagoya City University, Nagoya, Japan; Albert-Ludwigs-University, Germany

## Abstract

To find histone deacetylase 3 (HDAC3)-selective inhibitors, a series of 504 candidates was assembled using “click chemistry”, by reacting nine alkynes bearing a zinc-binding group with 56 azide building blocks in the presence of Cu(I) catalyst. Screening of the 504-member triazole library against HDAC3 and other HDAC isozymes led to the identification of potent and selective HDAC3 inhibitors **T247** and **T326**. These compounds showed potent HDAC3 inhibition with submicromolar IC_50_s, whereas they did not strongly inhibit other isozymes. Compounds **T247** and **T326** also induced a dose-dependent selective increase of NF-κB acetylation in human colon cancer HCT116 cells, indicating selective inhibition of HDAC3 in the cells. In addition, these HDAC3-selective inhibitors induced growth inhibition of cancer cells, and activated HIV gene expression in latent HIV-infected cells. These findings indicate that HDAC3-selective inhibitors are promising candidates for anticancer drugs and antiviral agents. This work also suggests the usefulness of the click chemistry approach to find isozyme-selective HDAC inhibitors.

## Introduction

Histone protein complexes associate with DNA to form higher-order structures called chromatin. Approximately 150 base pairs of DNA are wrapped twice around an octamer of histones to form a nucleosome, the basic unit of chromatin. Core histones with *N*-terminal tails extending from the compact nucleosomal core particles can be acetylated or deacetylated at the epsilon position of lysine residues, thereby modifying histone-DNA and histone-non-histone protein interactions. The acetylation status of histone and non-histone proteins is controlled by two enzyme classes with opposing activities; histone acetyltransferases and histone deacetylases (HDACs) [1]–[3]. HDACs are hydrolases that modulate epigenetic gene expression through deacetylation of the *N*-acetyl lysine residues of histone and non-histone proteins. There are currently 18 known HDACs that are organized into four classes: class I HDACs (HDAC1, HDAC2, HDAC3, and HDAC8) and class IV HDAC (HDAC11) which are mainly localized to the nucleus; class II HDACs (HDAC4, HDAC5, HDAC6, HDAC7, HDAC9, and HDAC10) which shuttle between the nucleus and the cytoplasm; and class III HDACs (sirtuin 1–7), whose cellular localizations include various organelles [4]. Class I, II, IV HDACs are zinc-dependent enzymes, whereas class III HDACs are NAD^+^-dependent enzymes [5]–[8].

Among the HDAC family members, HDAC3 is unique in that it is expressed in the nucleus, cytoplasm, or membrane, and it deacetylates histone and non-histone proteins such as NF-κB, myocyte enhancer factor 2, and Src kinase [9]–[16]. Furthermore, recent studies have indicated that HDAC3 is associated with several diseases including cancer, inflammation, and neurodegenerative disorders [17]–[20]. Therefore, HDAC3-selective inhibitors are of great interest not only as tools for probing the biological functions of HDAC3, but also as candidate therapeutic agents with potentially few side effects.

**Figure 1:**
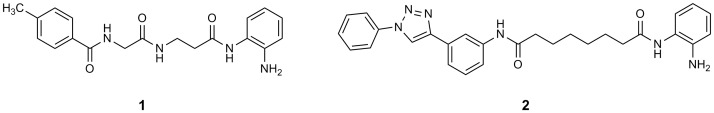
Previously reported HDAC3-selective inhibitors 1 and 2.

Although many efforts have been directed to the discovery of potent and selective HDAC inhibitors by numerous academic groups, as well as pharmaceutical companies, only a few HDAC3-selective inhibitors have been reported [4] [21]–[26]. For example, HDAC3 is selectively inhibited by compounds **1** and **2** (Figure 1) [27]–[28], but their HDAC3-inhibitory activity and selectivity are insufficient for their development as candidate therapeutic agents. In addition, while this research was carried out, RGFP966, a novel HDAC3-selective inhibitor, was reported, although the details of the inhibitor are unclear [29]. Therefore, there is still a need to find HDAC3 inhibitors that are more potent and selective than compounds **1** and **2**.

We recently described the identification of potent HDAC8-selective inhibitors from a triazole compound library generated by the use of Cu(I)-catalyzed azide-alkyne cycloaddition (CuAAC), a representative reaction in click chemistry [30]–[33]. Our results indicated that the click chemistry approach is useful for the discovery of isozyme-selective HDAC inhibitors. Following these findings, we performed a further click chemistry approach, seeking to find HDAC3-selective inhibitors more potent and selective than compounds **1** and **2**. We describe here the rapid identification of potent and selective HDAC3 inhibitors via the use of click chemistry to generate a library of HDAC inhibitor candidates.

## Results and Discussion

### Enzyme Assays

**Figure 2:**
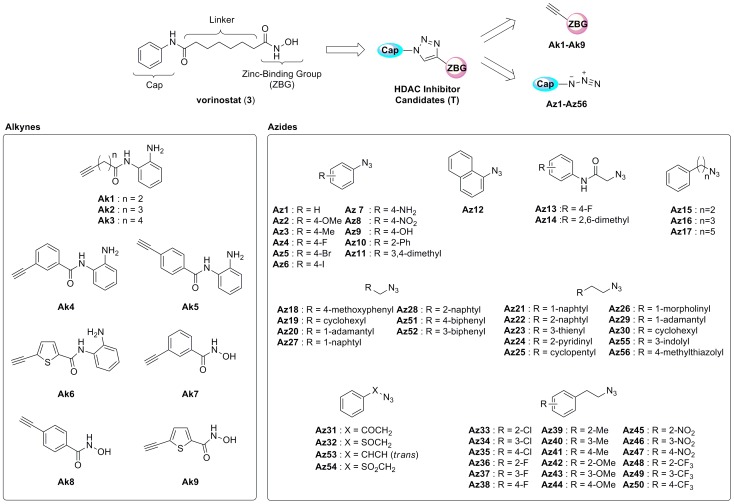
Design of triazole-containing HDAC inhibitor candidates.

Most HDAC inhibitors reported so far fit a three-motif pharmacophoric model, namely, a zinc-binding group (ZBG), a linker, and a cap group [21]–[26]. For instance, vorinostat (**3**) (Figure 2) [34] [35], a clinically used HDAC inhibitor, consists of hydroxamic acid (ZBG), which chelates the zinc ion in the active site, anilide (cap), which interacts with amino acid residues on the rim of the active site, and alkyl chain (linker), which connects the cap group and ZBG with an appropriate separation. Based on the typical HDAC inhibitor structure, we previously designed a library of candidate HDAC inhibitors in which the cap group and the ZBG are connected by a triazole-containing linker (Figure 2), and we identified potent HDAC8-selective inhibitors through screening of the library [30]. Following these findings, we expanded the library by the design and preparation of new alkynes with a ZBG and azides with a cap structure to find potent and selective HDAC3 inhibitors. For the preparation of the triazole library in this work, we designed and synthesized three alkynes **Ak1**–**Ak3** with *o*-aminoanilide as the ZBG and 14 azides **Az1**–**Az14** with an aromatic cap structure as building blocks for HDAC inhibitor candidate synthesis via CuAAC reaction. In designing alkynes **Ak1**–**Ak3**, *o*-aminoanilide was selected as the ZBG because *o*-aminoanilides tend to inhibit Class I HDACs [4]. Azides **Az1**–**Az14** bearing an aromatic ring were expected to interact with aromatic amino acid residues such as Tyr and Phe which form the HDAC3 active pocket [36].

**Figure 3:**
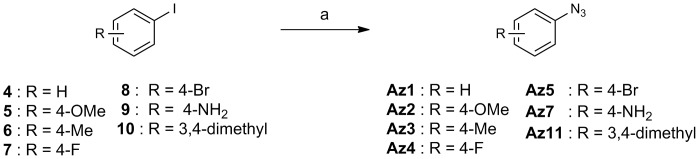
Scheme for the synthesis of Az1–Az5, Az7, and Az11. Reagents and conditions: (a) NaN_3_, CuI, l-Pro, NaOH, DMSO, 60°C, 37–95%.

**Figure 4:**
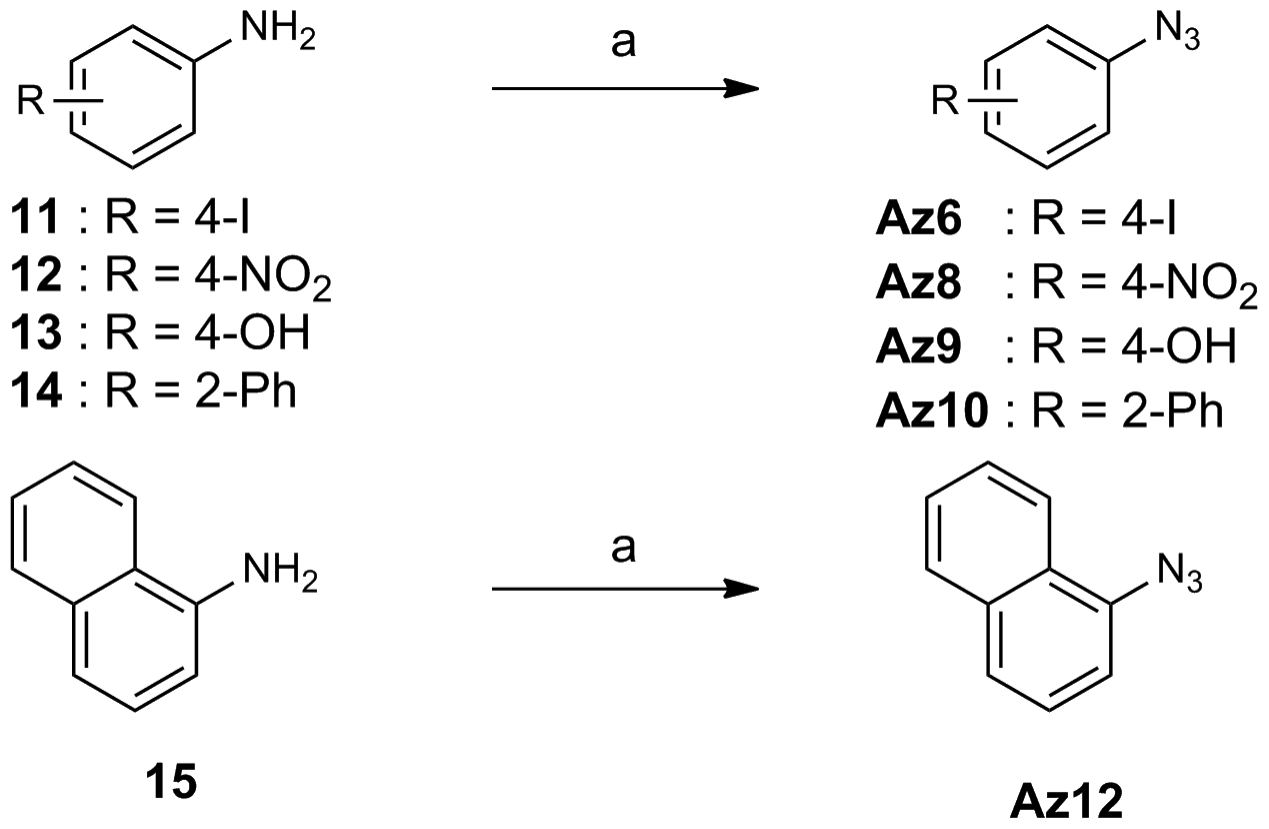
Scheme for the synthesis of Az6, Az8–Az10, and Az12. Reagents and conditions: (a) i) NaNO_2_, H_2_O, TFA, 0°C; ii) NaN_3_, H_2_O, 0°C to room temp, 18–90%.

**Figure 5:**
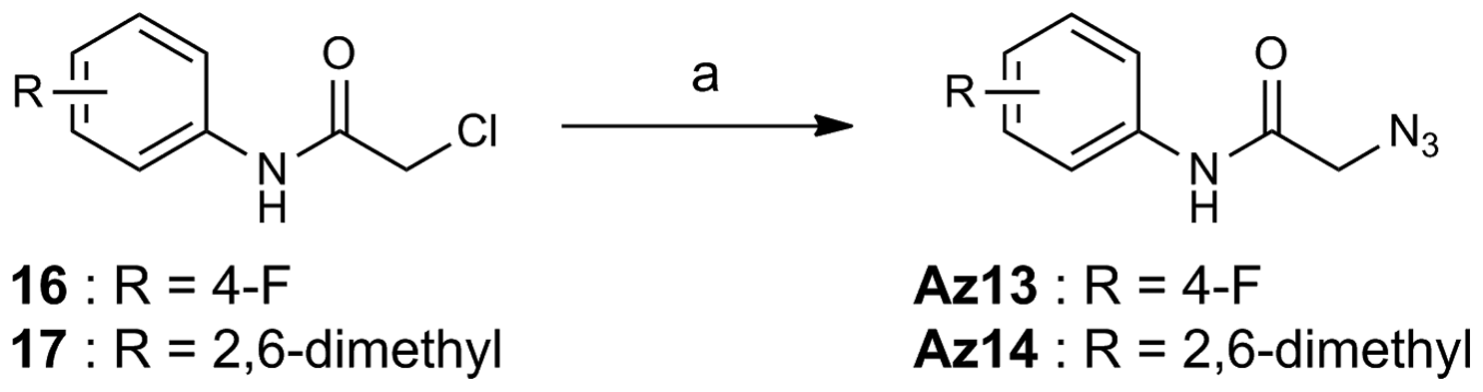
Scheme for the synthesis of Az13 and Az14. Reagents and conditions: (a) NaN_3_, DMSO, room temp, 97% for **Az13**; 64% for **Az14**.

**Figure 6:**
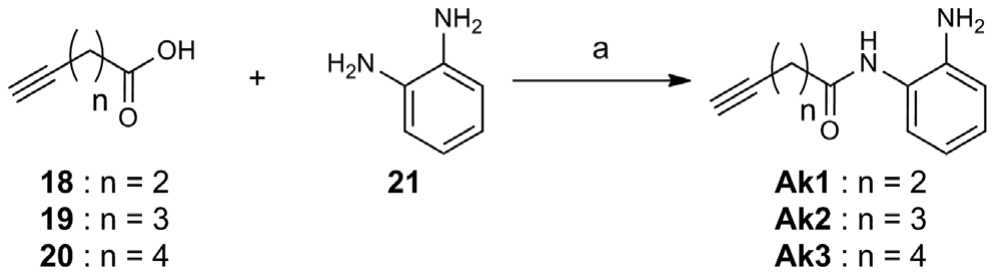
Scheme for the synthesis of Ak1–Ak3. Reagents and conditions: (a) EDCI, HOBt, DMF, room temp, 36–62%.

The routes used for the synthesis of compounds **Az1–Az14**, and **Ak1–Ak3**, which were prepared for this study, are shown in Figures 3, 4, 5, 6. Figure 3 shows the preparation of aryl azides **Az1–Az5**, **Az7**, and **Az11**. The coupling reaction of aryl iodides **4–10** with sodium azide was carried out in the presence of CuI/l-proline catalyst to provide aryl azides **Az1–Az5**, **Az7**, and **Az11** in 37–95% yield [37]. The routes for the synthesis of aryl azides **Az6**, **Az8–Az10**, and **Az12** are illustrated in Figure 4. Treatment of anilines **11–15** with NaNO_2_ under acidic conditions, followed by NaN_3_ addition, yielded the desired aryl azides **Az6**, **Az8–Az10**, and **Az12**. The preparation of alkyl azides **Az13** and **Az14** is shown in Figure 5. Chlorides **16** and **17** were allowed to react with NaN_3_ to afford alkyl azides **Az13** and **Az14**. Figure 6 shows the preparation of alkynes **Ak1–Ak3** bearing an *o*-aminoanilide moiety. Condensation of phenylenediamine **21** with the appropriate carboxylic acid chloride **18–20** gave *o*-aminoanilide derivatives **Ak1–Ak3**.

**Figure 7:**
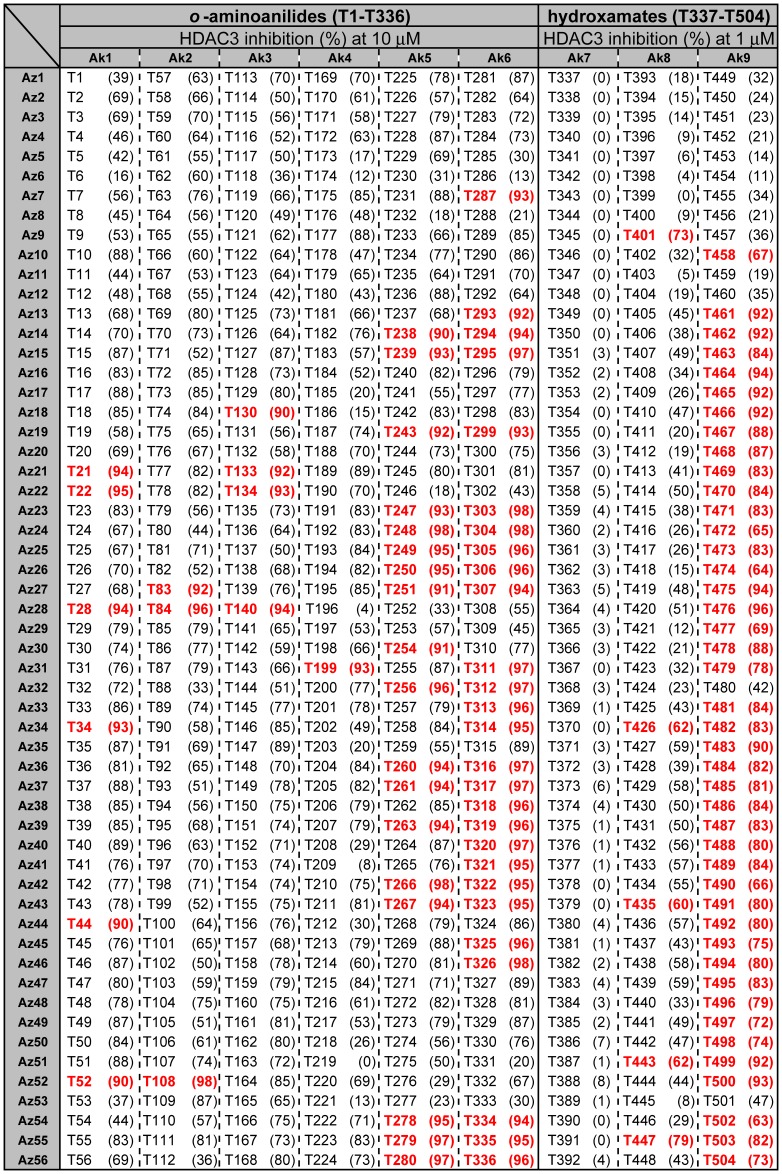
Inhibition of HDAC3 in the presence of T1–T504 (10 µM for *o*-aminoanilides T1–T336; 1 µM for hydroxamates T337–T504). *o*-Aminoanilides inhibiting more than 90% of HDAC3 activity and hydroxamates inhibiting more than 60% of HDAC3 activity are indicated in red. Vorinostat (**3**) (1 µM) and compound **1** (10 µM) inhibited 98% and 47% of HDAC3 activity, respectively.

The CuAAC reaction between nine alkynes (newly prepared **Ak1**–**Ak3** and previously prepared **Ak4**–**Ak9**) and 56 azides (newly prepared **Az1**–**Az14** and previously prepared **Az15**–**56**) allowed us to assemble a 504-member HDAC inhibitor candidate library in microtiter plates [30]–[38]. Alkynes **Ak1**–**Ak9** (1 eq) and azides **Az1**–**Az56** (1.4 eq) in the presence of CuSO_4_ (0.2 eq), sodium ascorbate (1 eq), and tris[(1-benzyl-1*H*-1,2,3-triazol-4-yl)methyl]amine (TBTA) (0.2 eq) in a solvent mixture of DMSO/H_2_O (1∶1) afforded the 504-membered triazole library. In all cases, disappearance of the alkynes and generation of the triazoles were confirmed by TLC. The generated triazole-containing HDAC inhibitor candidates **T1**–**T504** are shown in Figure 7.

**Figure 8:**
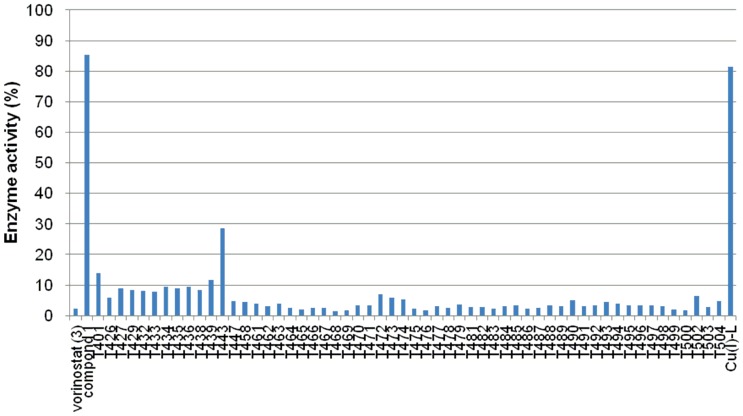
Total HDACs activity in the presence of 48 hydroxamates (1 µM).

**Figure 9:**
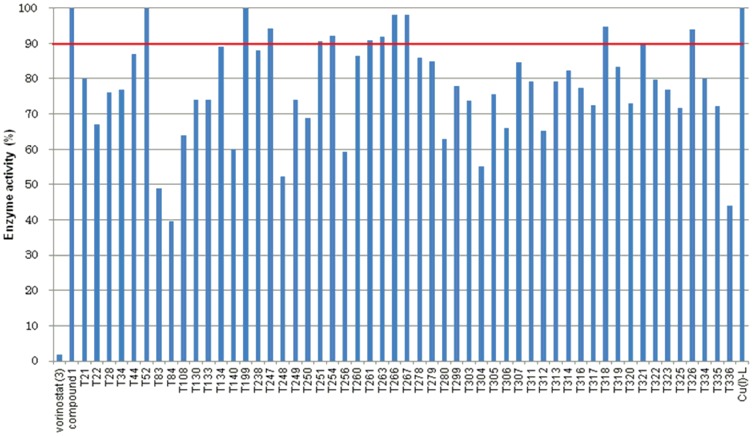
Activity of total HDACs in the presence of 59 *o*-aminoanilides (10 µM).

These triazole compounds could be screened for HDAC-inhibitory activity without further purification [30] [39]–[44]. Since our final goal in this work is to identify compounds that selectively inhibit HDAC3 in cells, it is desirable to carry out *in vitro* enzyme assays in conditions similar to cellular environments. Because HDAC3 forms a complex with NCOR1 in cells [45], we used HDAC3/NCOR1 complex in *in vitro* HDAC3 assay. In addition, it is more important to find inhibitors that discriminate HDAC3 from HDAC1 and HDAC2 in cells. Therefore, as a primary *in vitro* screening for HDAC3 selectivity, we used total HDACs from HeLa nuclear extracts, in which the combined deacetylase activity of HDAC1 and HDAC2 is much higher than the activity of HDAC3 [46]. Initially, *o*-aminoanilides **T1**–**T336** (10 µM) and hydroxamates **T337**–**T504** (1 µM) were tested for inhibitory activity against HDAC3. In our HDAC3 assay, the IC_50_ values of compounds **1**–**3** were 19 µM, >100 µM, and 0.27 µM, respectively. We therefore used compound **1** and vorinostat (**3**) as reference compounds in this assay. As shown in Figure 7, 59 *o*-aminoanilides inhibited HDAC3 deacetylase activity by more than 90% at 10 µM, and 48 hydroxamates showed more than 60% HDAC3 inhibition at 1 µM. Next, we evaluated these 107 compounds for inhibitory activity against total HDACs from HeLa nuclear extracts, in which the deacetylase activity of HDAC1 and HDAC2 is much higher than that of HDAC3 [46]. While all of the hydroxamates displayed more than 70% inhibition of total HDACs at 1 µM (Figure 8), 11 *o*-aminoanilides showed less than 10% inhibition at 10 µM (Figure 9) suggesting that these *o*-aminoanilides exhibited HDAC3-selective inhibition. Furthermore, we investigated the HDAC3-inhibitory activity of these 11 *o*-aminoanilides at 1 µM and 3 µM. Among them, **T247** and **T326** showed HDAC3 inhibition comparable to that of vorinostat (**3**) at both 1 µM and 3 µM (Table 1). These results indicated that **T247** and **T326** might be potent and selective HDAC3 inhibitors.

**Table 1:** HDAC3 inhibition in the presence of vorinostat (3), compound 1, and 11 o-aminoanilides at 1 µM and 3 µM.^a^

Conc.	HDAC3 inhibition (%)												
	3	1	T52	T199	T247	T251	T254	T261	T263	T266	T267	T318	T326
1 µM	83	9	55	59	89	75	55	75	74	73	80	77	86
3 µM	93	29	81	83	95	92	80	89	91	91	92	91	95

a Values are means of two experiments.

**Figure 10:**
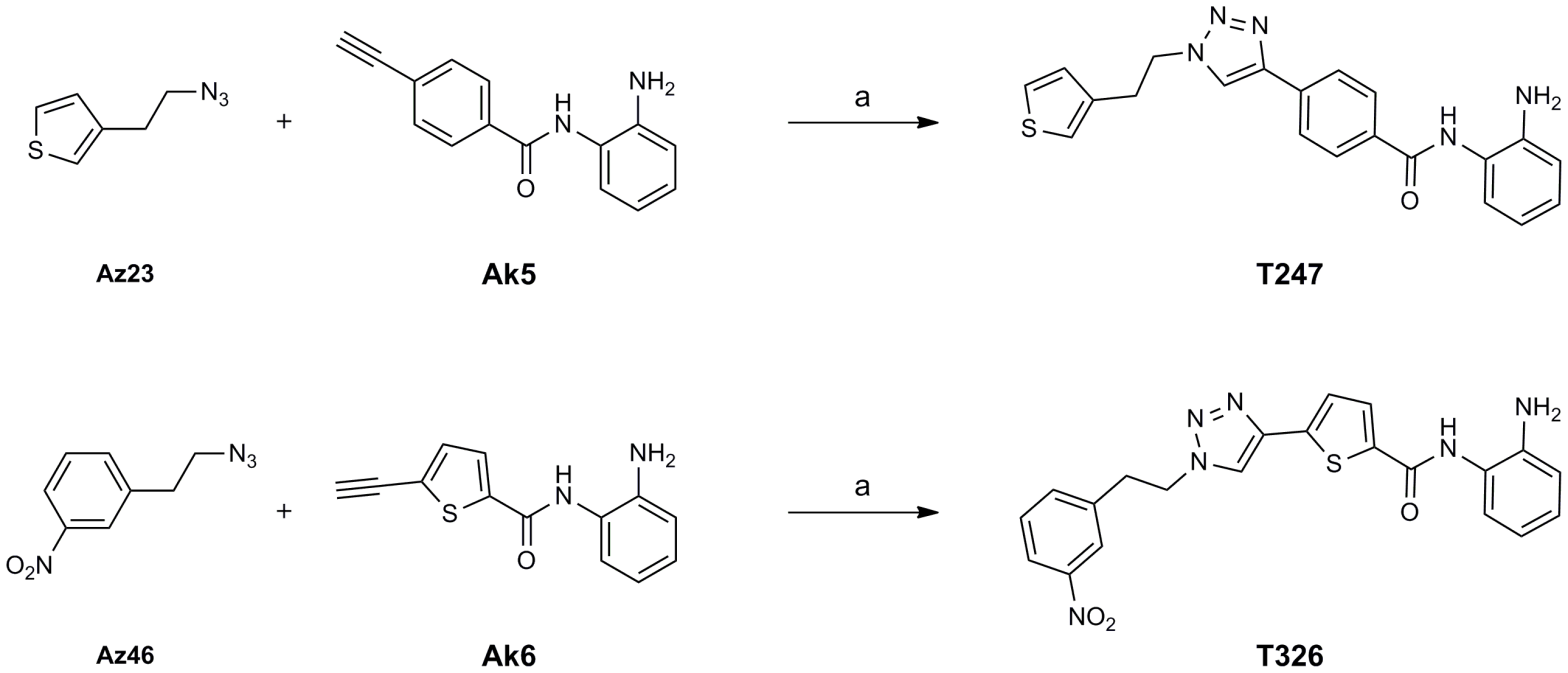
Scheme for the synthesis of T247 and T326. Reagents and conditions: (a) CuSO_4_, sodium ascorbate, EtOH, H_2_O, room temp, 65% for **T247**; 97% for **T326**.

Figure 10 illustrates the resynthesis of triazoles **T247** and **T326**. Cu-catalyzed coupling of alkyne **Ak5** with **Az23** and **Ak6** with **Az46** provided triazoles **T247** and **T326**, respectively. The resynthesized compounds **T247** and **T326** were purified by column chromatography and recrystallization. The pure **T247** and **T326** were then examined for inhibitory effects on total HDACs, HDAC1, HDAC4, HDAC6, and HDAC8. The results of the enzyme assays are shown in Table 2. Compounds **T247** and **T326** displayed potent HDAC3-inhibitory activity, greater than that of compound **1** and comparable to that of vorinostat (**3**) (IC_50_ of **1** 19 µM, vorinostat (**3**) 0.27 µM, **T247** 0.24 µM, **T326** 0.26 µM). Furthermore, while vorinostat (**3**) inhibited total HDACs, HDAC1, HDAC6, and HDAC8, compounds **T247** and **T326** inhibited HDAC3 selectively over the other isozymes. Thus, **T247** and **T326** are potent and selective inhibitors of HDAC3.

**Table 2:** HDAC-Inhibitory Activity of vorinostat (3), compound 1, T247, and T326 a.

Compound	IC_50_ (µM)					
	class I				class IIa	class IIb
	HDACs	HDAC1	HDAC3	HDAC8	HDAC4	HDAC6
**vorinostat (3)**	0.073	0.39	0.27	0.66	>10	0.34
**1**	>100	>100	19	>100	>100	>100
**T247**	>100	19	0.24	>100	>100	>100
**T326**	>100	>100	0.26	>100	>100	>100

a Values are means of at least three experiments.

### Molecular Modeling

**Figure 11:**
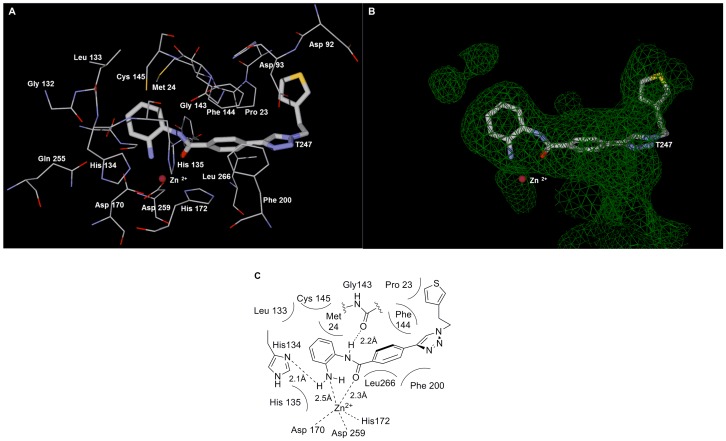
Binding mode of T247. (A) View of the conformation of **T247** (tube) docked in the HDAC3 catalytic core. Compound **T247** was docked into a model based on the crystal structure of HDAC3 (PDB code 4A69) using the Molegro Virtual Docker software package. Residues around **T247** are displayed as wires. (B) The same view as A. The narrow and long tunnel of the active site is displayed as a green mesh. (C) Schematic diagram of **T247**-binding to the catalytic site.

The lowest energy conformation of **T247**, the most active HDAC3-selective inhibitor in this series, was obtained when it was docked into a model based on the crystal structure of HDAC3 (PDB code 4A69) [36], using the Molegro Virtual Docker software package. Inspection of the simulated HDAC3/**T247** complex showed that the *o*-aminoanilide group coordinates to the Zn ion bidentately through its NH_2_ and CO groups, and also forms two hydrogen bonds with His 134 and Gly 143 (Figure 11). In addition, the phenyltriazole part of the inhibitor snugly fits the catalytic site. The phenyltriazole group of **T247** lies in the hydrophobic tunnel formed by Phe 144, Phe 200, and Leu 266, where it can interact with the amino acid residues via hydrophobic interactions. There also appears to be a hydrophobic interaction of the thiophene ring of **T247** with Pro 23 and Phe 144. The observed interactions between **T247** and HDAC3 suggest the importance of the *o*-aminoanilide as a ZBG and a hydrogen-bond-forming group for high potency. They also suggest the significance of the lipophilic aromatic rings of **T247** for hydrophobic interactions. The triazole ring appears to orient the ZBG and hydrophobic group into appropriate geometry.

### Cell-based Assays

**Figure 12:**
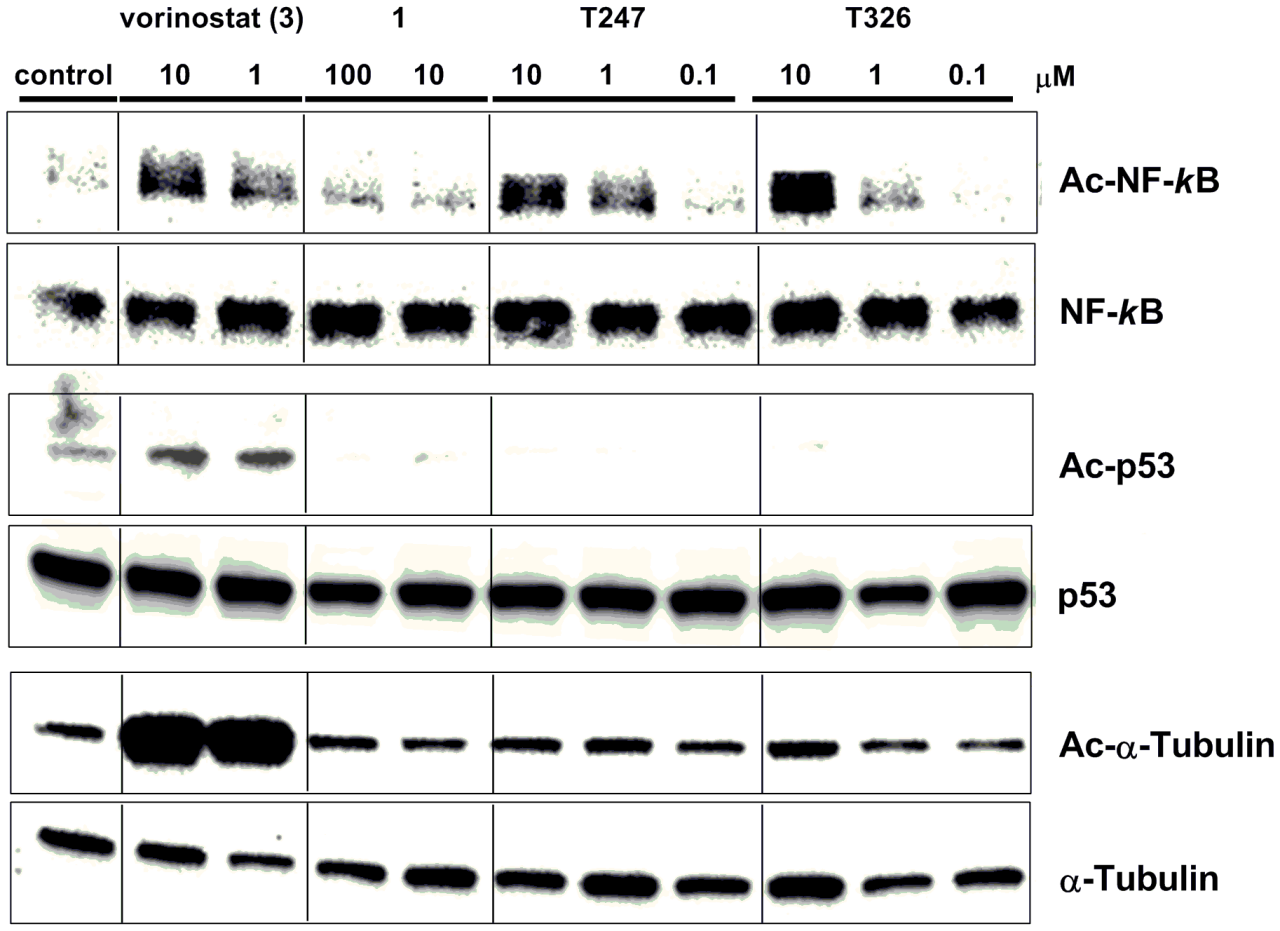
Western blot detection of acetylated NF-*κ*B, p53, and α-tubulin levels in HCT116 cells after 8 h treatment with vorinostat (3), compound 1, T247, and T326.

To examine whether compounds **T247** and **T326** selectively inhibit HDAC3 in cells, we performed a cellular assay using western blot analysis. Since HDAC3 is known to catalyze the deacetylation of NF-κB [13]–[14], we initially examined the effects of the inhibitors on the acetylation levels of NF-κB in HCT116 cells. As we expected, **T247** and **T326** induced a dose-dependent increase of NF-κB acetylation, and their effect was greater than that of compound **1** and comparable to that of vorinostat (**3**) (Figure 12). Although **T247** and **T326** caused NF-κB acetylation, it has also been reported that NF-κB is deacetylated by HDAC1 and HDAC2 [47]. To examine whether **T247** and **T326** can distinguish HDAC3 from HDAC1 in cells, we next analyzed the effects of **T247** and **T326** on the acetylation levels p53, a substrate protein of HDAC1 [48]. As can be seen in Figure 12, while vorinostat (**3**), a non-selective HDAC inhibitor, induced non-selective acetylation of NF-κB and p53, the levels of acetylated p53 were not elevated in the presence of **T247** and **T326**. These results indicate that **T247** and **T326** do not inhibit HDAC1 and selectively inhibit HDAC3 in the cells. In addition, **T247** and **T326** did not enhance the acetylation of α-tubulin, a substrate of HDAC6 [49] suggesting that **T247** and **T326** are HDAC3-selective inhibitors in cell-based assays.

Because it has been suggested that HDAC3 is highly expressed in human colon cancer cells and prostate cancer cells and is associated with the cancer cell growth [50]–[51], vorinostat (**3**), compound **1**, **T247**, and **T326** were tested in cell growth-inhibition assays using human colon cancer HCT116 and prostate cancer PC-3 cell lines. The results are shown in Table 3. HDAC3-selective inhibitors **T247** and **T326** showed clear growth-inhibitory effects on both HCT116 and PC-3 cell lines. In particular, the cell growth-inhibitory activity of compound **T247** and **T326** was much greater than that of compound **1** and comparable to that of vorinostat (**3**). These results suggest that HDAC3-selective inhibitors might be useful in the treatment of colon cancers and prostate cancers.

**Table 3:** Growth inhibition of colon cancer HCT116 cells and prostate cancer PC3 cells by vorinostat (3), compound 1, T247, and T326^a^.

Cell line	GI_50_ (µM)			
	3	1	T247	T326
HCT116 (colon cancer)	1.3	81	1.9	0.94
PC3 (prostate cancer)	1.6	>100	1.4	1.0

a Values are means of at least three experiments.

**Figure 13:**
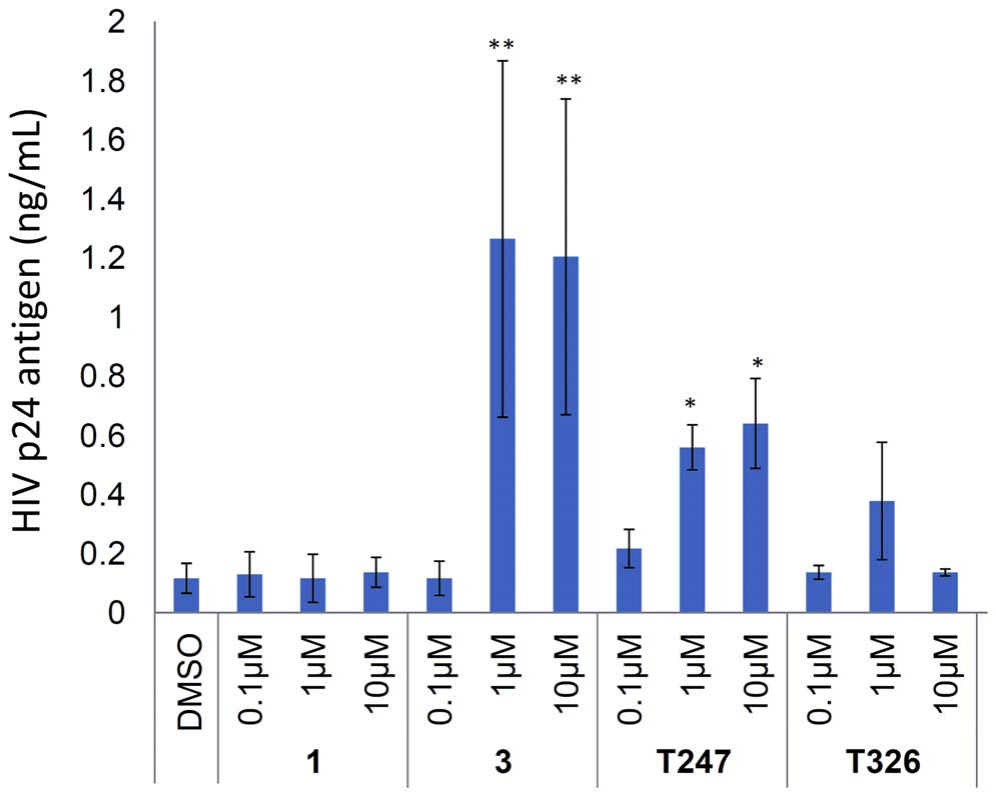
Induction of viral replication from OM10.1 cells latently infected with HIV-1. Cells were incubated with compound **1**, vorinostat (**3**), **T247**, and **T326** for 48 h. HIV-1 p24 antigen in the cell culture supernatant was measured using ELISA. Experiments were performed in triplicate, and the means ±S.D. are indicated. **P<0.01, *P<0.05; Student’s *t* test results indicated differences between DMSO and inhibitors.

We also examined the effects of **T247** and **T326** on latent HIV-infected cells, because it has been suggested that HDAC3 represses the transcription of HIV type 1 (HIV-1) genes in such cells [52]. HIV-1-infected OM10.1 cells were treated with 0.1 µM, 1 µM, and 10 µM compound **1**, vorinostat (**3**), **T247**, and **T326**. Although compound **1**, a weak HDAC3 inhibitor, did not show any activity, vorinostat (**3**), **T247**, and **T326** significantly stimulated HIV-1 expression at 1 µM and/or 10 µM (Figure 13). Compound **T326** was less active at 10 µM due to cytotoxicity. These data suggest that the combination of HDAC3-selective inhibitor and other anti-HIV agents may be useful in the treatment of HIV infection [53]–[55].

In summary, we have designed a 504-membered triazole-containing HDAC inhibitor candidate library and prepared it by means of CuAAC reaction between nine alkynes and 56 azides. Two compounds, **T247** and **T326**, were hit as HDAC3-selective inhibitors by screening of the 504 library compounds. Compounds **T247** and **T326** showed potent inhibition of HDAC3 with IC_50_ values of 0.24 µM and 0.26 µM, respectively, but did not inhibit other HDAC isozymes even at 100 µM. The molecular modeling study of **T247** with HDAC3 suggested the importance of the *o*-aminoanilide as a ZBG and a hydrogen-bond-forming group, and of the lipophilic part having three aromatic rings for hydrophobic interactions. In cellular assays, **T247** and **T326** induced a selective increase of acetylated NF-κB, suggesting that they are cellularly active HDAC3-selective inhibitors. **T247** and **T326** also inhibited the growth of colon cancer HCT116 and prostate cancer PC-3 cell lines, and stimulated HIV-1 gene expression in latent HIV-1-infected OM10.1 cells. We believe that **T247** and **T326** are the most potent HDAC3-selective inhibitors reported so far. The findings presented here should provide a basis for constructing new tools to probe the biology of HDAC3 and for developing new strategies to treat cancer and HIV-1 infection.

Many groups have ongoing research programs to find selective inhibitors of HDAC isozymes, however, there has been no reported isozyme-selective inhibitors of HDAC1, 2, 5, 7, 9, 10, and 11, although the isozymes have been reported to be crucial for biological events and be responsible for several disease states [4]. Our methodology using click chemistry could be used to find not only HDAC3- and HDAC8-selective inhibitors, but also other isozyme-selective inhibitors. We believe that selective inhibitors against the HDAC isozymes will be discovered using this click chemistry approach in the near future.

## Materials and Methods

### Chemistry

#### General

Melting points were determined using a Yanagimoto micro melting point apparatus or a Büchi 545 melting point apparatus and were left uncorrected. Proton nuclear magnetic resonance spectra (^1^H NMR), carbon nuclear magnetic resonance spectra (^13^C NMR) were recorded on a JEOL JNM-LA500, JEOL JNM-A500 or BRUKER AVANCE600 spectrometer in the indicated solvents. Chemical shifts (δ) are reported in parts per million relative to the internal standard tetramethylsilane. Elemental analysis was performed with a Yanaco CHN CORDER NT-5 analyzer, and all values were within ±0.4% of the calculated values. Fast atom bombardment (FAB) mass spectra were recorded on a JEOL JMS-SX102A mass spectrometer. GC-MS analyses were performed on a Shimadzu GCMS-QP2010. IR spectra were measured on a Shimadzu FTIR-8400S spectrometer. Reagents and solvents were purchased from Aldrich, Tokyo Kasei Kogyo, Wako Pure Chemical Industries, and Kanto Kagaku and used without purification. Flash column chromatography was performed using silica gel 60 (particle size 0.046–0.063 mm) supplied by Merck.

### Synthesis

#### Azidobenzene (Az1)

A mixture of iodobenzene (**4**, 0.33 mL, 3.0 mmol), CuI (57 mg, 0.30 mmol), l-proline (69 mg, 0.60 mmol), and a 0.5 M solution of NaN_3_ in DMSO (12 mL, 6.0 mmol) was stirred at 60°C for 19 h and then allowed to cool to room temperature. The reaction mixture was diluted with AcOEt, washed with water and brine, and dried over Na_2_SO_4_. Filtration, concentration in vacuo, and purification by silica gel flash column chromatography (*n*-hexane only) gave 293 mg (82%) of **Az1** as a yellow oil. ^1^H NMR (DMSO-*d*_6_, 500 MHz, δ, ppm) 7.42 (2H, t, *J* = 7.9 Hz), 7.20 (1H, t, *J* = 7.5 Hz), 7.12 (1H, d, *J* = 7.5 Hz). FTIR (neat, cm^−1^) 2091. MS (EI) m/z 119 (M^+^).

Compounds **Az2–Az5**, **Az7**, and **Az11** were prepared from an appropriate iodobenzene (**5–10**) and NaN_3_ using the procedure described for **Az1**.

#### 1-Azido-4-methoxybenzene (Az2)

Yield 77%; white solid; ^1^H NMR (DMSO-*d*_6_, 500 MHz, δ, ppm) 7.06 (2H, d, *J* = 8.8 Hz), 6.98 (2H, d, *J* = 8.8 Hz), 3.74 (1H, s). FTIR (neat, cm^−1^) 2106. MS (EI) m/z 149 (M^+^).

#### 1-Azido-4-methylbenzene (Az3)

Yield 41%; yellow oil; ^1^H NMR (DMSO-*d*_6_, 500 MHz, δ, ppm) 7.22 (2H, d, *J* = 7.5 Hz), 7.01 (2H, d, *J* = 8.5 Hz), 2.28 (3H, s). FTIR (neat, cm^−1^) 2121. MS (EI) m/z 133 (M^+^).

#### 1-Azido-4-fluorobenzene (Az4)

Yield 50%; yellow oil; ^1^H NMR (DMSO-*d*_6_, 500 MHz, δ, ppm) 7.31–7.22 (2H, m), 7.21–7.13 (2H, m). FTIR (neat, cm^−1^) 2106. MS (EI) m/z 137 (M^+^).

#### 1-Azido-4-bromobenzene (Az5)

Yield 54%; yellow oil; ^1^H NMR (DMSO-*d*_6_, 500 MHz, δ, ppm) 7.60 (1H, d, *J* = 9.0 Hz), 7.11 (2H, d, *J* = 9.0 Hz). FTIR (neat, cm^−1^) 2121. MS (EI) m/z 197 (M^+^), 199 (M^+^+2).

#### 4-Azidoaniline (Az7)

Yield 37%; red solid; ^1^H NMR (DMSO-*d*_6_, 500 MHz, δ, ppm) 6.77 (2H, d, *J* = 8.7 Hz), 6.59 (2H, d, *J* = 8.8 Hz), 5.13 (2H, s); FTIR (neat, cm^−1^) 2106. MS (EI) m/z 134 (M^+^).

#### 1-Azido-3,4-dimethylbenzene (Az11)

Yield 95%; yellow oil; ^1^H NMR (DMSO-*d*_6_, 500 MHz, δ, ppm) 7.18 (1H, d, *J* = 8.0 Hz), 6.92 (1H, s), 6.84 (1H, d, *J* = 8.0 Hz). FTIR (neat, cm^−1^) 2102. MS (EI) m/z 147 (M^+^).

#### 1-Azido-4-iodobenzene (Az6)

To a solution of 4-iodoaniline (**11**, 1.07 g, 4.87 mmol) in TFA (10 mL) was added a solution of NaNO_2_ (1.45 g, 21.0 mmol) in water (10 mL) at 0°C. The mixture was stirred at 0°C for 10 min and a solution of NaN_3_ (3.2 g, 49.2 mmol) in water (10 mL) was added. The reaction mixture was diluted with AcOEt, washed with water and brine, and dried over Na_2_SO_4_. Filtration and concentration in vacuo, and recrystallization from AcOEt gave 1.07 g (90%) of **Az6** as a black solid. ^1^H NMR (DMSO-*d*_6_, 500 MHz, δ, ppm) 7.73 (2H, d, *J* = 8.5 Hz), 6.95 (2H, d, *J* = 8.5 Hz). FTIR (neat, cm^−1^) 2096. MS (EI) m/z 245 (M^+^).

Compounds **Az8–Az10** and **Az12** were prepared from an appropriate aniline (**11–15**) using the procedure described for **Az6**.

#### 4-Azidonitrobenzene (Az8)

Yield 80%; yellow solid; ^1^H NMR (DMSO-*d*_6_, 500 MHz, δ, ppm) 8.24 (2H, d, *J* = 9.0 Hz), 7.35 (2H, d, *J* = 9.0 Hz). FTIR (neat, cm^−1^) 2121. MS (EI) m/z 164 (M^+^).

#### 4-Azidophenol (Az9)

Yield 18%; black solid; ^1^H NMR (DMSO-*d*_6_, 500 MHz, δ, ppm) 9.55 (1H, s), 6.91 (2H, d, *J* = 9.0 Hz), 6.78 (2H, d, *J* = 9.0 Hz); FTIR (CHCl_3_, cm^−1^) 2114; MS (EI) m/z 135 (M^+^).

#### 2-Azidophenylbenzene (Az10)

Yield 87%; yellow oil; ^1^H NMR (DMSO-*d_6_*, 500 MHz, δ, ppm) 7.50–7.40 (5H, m), 7.37 (3H, t, *J* = 8.0 Hz), (1H, t, *J* = 7.3 Hz). FTIR (CHCl_3_, cm^−1^) 2125. MS (EI) m/z 167 (M^+^–N_2_).

#### 1-Azidonaphthalene (Az12)

Yield 43%; brown oil; ^1^H NMR (DMSO-*d*_6_, 500 MHz, δ, ppm) 7.94 (1H, d, *J* = 8.0 Hz), 7.87 (1H, d, *J* = 8.0 Hz), 7.68 (1H, d, *J* = 8.0 Hz), 7.53–7.44 (3H, m), 7.36 (1H, d, *J* = 7.5 Hz). FTIR (neat, cm^−1^) 2110. MS (EI) m/z 169 (M^+^).

#### 2-Azido-*N*-(4-fluorophenyl)acetamide (Az13)

To a solution of 0.5 M NaN_3_ (16 mmol) in DMSO (32 mL) was added 2-chloro-*N*-(4-fluorophenyl)acetamide (**16**, 1.0 g, 5.3 mmol), and the mixture was stirred at room temperature for 24 h. The reaction mixture was diluted with AcOEt, washed with water and brine, and dried over Na_2_SO_4._ Filtration, concentration in vacuo, and purification by silica gel flash column chromatography (AcOEt/*n*-hexane = 1/2) gave 1.0 g (97%) of **AZ13** as a brown solid. ^1^H NMR (DMSO-*d*_6_,_,_ 500 MHz, δ, ppm) 10.2 (1H, s), 7.60–7.55 (2H, m), 7.19–7.12 (2H, m), 4.03 (2H, s). FTIR (neat, cm^−1^) 2102. MS (EI) m/z 194 (M^+^).

Compound **Az14** was prepared from 2-chloro-*N*-(2,6-dimethylphenyl)acetamide **17** and NaN_3_ using the procedure described for **Az13**.

#### 2-Azido-*N*-(2,6-dimethylphenyl)acetamide (Az14)

Yield 64%; white solid; ^1^H NMR (DMSO-*d*_6,_ 500 MHz, δ, ppm) 9.51 (1H, s), 7.09 (3H, m), 4.09 (2H, s), 2.14 (6H, s). FTIR (neat, cm^−1^) 2094. MS (EI) m/z 176 (M^+^−N_2_).

#### Pent-4-ynoic acid (2-aminophenyl)amide (Ak1)

A mixture of 4-pentynoic acid (**18**, 437 mg, 4.45 mmol), 1,2-phenylenediamine (**21**, 407 mg, 3.76 mmol), EDCI (874 mg, 4.56 mmol), and HOBt·H_2_O (629 mg, 4.65 mmol) in dry DMF was stirred at room temperature for 6 h. The reaction mixture was diluted with AcOEt, washed with water and brine, and dried over Na_2_SO_4._ Filtration, concentration in vacuo, and purification by silica gel flash column chromatography (AcOEt/*n*-hexane = 1/1) gave 400 mg (56%) of **AK1** as a white solid. ^1^H NMR (CD_3_OD, 500 MHz, δ, ppm) 7.07 (1H, d, *J* = 8.0 Hz), 7.02 (1H, t, *J* = 7.5 Hz), 6.83 (1H, d, *J* = 7.8 Hz), 6.70 (1H, t, *J* = 7.5 Hz), 2.63–2.57 (4H, m), 2.34–2.33 (1H, m). MS (EI) m/z 188 (M^+^).

Compounds **Ak2** and **Ak3** were prepared from an appropriate carboxylic acid (**19** or **20**) and 1,2-phenylenediamine **21** using the procedure described for **Ak1**.

#### Hex-5-ynoic acid (2-aminophenyl)amide (Ak2)

Yield 36%; pink solid; ^1^H NMR (CD_3_OD, 500 MHz, δ, ppm) 7.08 (1H, d, *J* = 7.8 Hz), 7.02 (1H, t, *J* = 7.5 Hz), 6.84 (1H, d, *J* = 8.0 Hz), 6.71 (1H, t, *J* = 7.5 Hz), 2.55 (2H, t, *J* = 7.5 Hz), 2.32–2.27 (3H, m), 1.91 (2H, quintet, *J* = 7.0 Hz). MS (EI) m/z 202 (M^+^).

#### Hept-6-ynoic acid (2-aminophenyl)amide (Ak3)

Yield 62%; pink solid; ^1^H NMR (CD_3_OD, 500 MHz, δ, ppm) 7.07 (1H, d, *J* = 7.8 Hz), 7.02 (1H, t, *J* = 7.8 Hz), 6.84 (1H, d, *J* = 8.3 Hz), 6.71 (1H, t, *J* = 7.8 Hz), 2.44 (2H, t, *J* = 7.5 Hz), 2.28–2.24 (3H, m), 1.83 (2H, quintet, *J* = 7.5 Hz) 1.62 (2H, quintet, *J* = 7.5 Hz). MS (EI) m/z 216 (M^+^).

#### Construction of Triazole Library (T1-T504)

To a solution of alkyne (25 mM, 20 µL), azide (35 mM, 20 µL), and TBTA (10 mM, 10 µL) in DMSO was added an aqueous solution of CuSO_4_·5H_2_O (4 mM, 25 µL) on a 96-well plate. To the resulting mixture was added an aqueous solution of sodium ascorbate (20 mM, 25 µL), and the mixture was shaken for 2–3 days at room temperature. Reactions were monitored by TLC. After the reactions were completed, the triazoles were diluted to desired concentrations for enzyme assays by adding DMSO.

#### *N*-(2-Aminophenyl)-4-[1-(2-thiophen-3-ylethyl)-1*H*-[1], [2], [3]triazol-4-yl]benzamide (T247)

A mixture of **Az23** (78 mg, 0.51 mmol), **Ak5** (65 mg, 0.28 mmol), CuSO_4_·5H_2_O (13.7 mg, 0.055 mmol), and sodium ascorbate (21.8 mg, 0.11 mmol) in water and EtOH (v/v = 1/1) was stirred vigorously for 15 h at room temperature. The reaction mixture was poured into water and extracted with AcOEt. The AcOEt layer was washed with brine, and dried over Na_2_SO_4_. Filtration, concentration in vacuo, and purification by silica gel flash column chromatography (AcOEt/*n*-hexane = 2/1) gave 70 mg (65%) of **T247** as a crude solid. The solid was recrystallized from water and MeOH to give 58 mg of **T247** as colorless crystals. mp 194–195°C. ^1^H NMR (DMSO-*d*_6_, 500 MHz, δ, ppm) 9.70 (1H, s), 8.66 (1H, s) 8.06 (2H, d, *J* = 8.0 Hz), 7.94 (2H, d, *J* = 8.5 Hz), 7.48 (1H, t, *J* = 3.0 Hz), 7.25 (1H, s), 7.17 (1H, d, *J* = 8.0 Hz), 7.02–6.95 (2H, m), 6.78 (1H, d, *J* = 8.0 Hz), 6.60 (1H, t, *J* = 8.0 Hz), 4.91 (2H, s), 4.68 (2H, t, *J* = 7.5 Hz), 3.25 (2H, d, *J* = 7.5 Hz). ^13^C NMR (DMSO-*d*_6_, 150 MHz, δ, ppm) 164.87, 145.42, 143.19, 137.76, 133.65, 128.53, 128.24, 127.00, 126.74, 126.53, 126.26, 125.49, 124.73, 122.19, 122.15, 116.27, 116.14, 50.09, 30.19. MS (FAB) *m/z* 390 (MH^+^). Anal. (C_21_H_19_N_5_OS) C, H, N.

Compound **T326** was prepared from **Az46** and **Ak6** using the procedure described for **T247**.

#### 5-{1-[2-(3-Nitrophenyl)ethyl]-1*H*-[1], [2], [3]triazol-4-yl}thiophene-2-carboxylic acid (2-aminophenyl)amide (T326)

Yield 97%; pale yellow crystals; mp 180–181°C. ^1^H NMR (DMSO-*d*_6_, 500 MHz, δ, ppm) 9.74 (1H, s), 8.56 (1H, s) 8.17 (1H, s), 8.10 (1H, d, *J* = 8.0 Hz), 7.96 (1H, m), 7.68 (1H, d, *J* = 7.0 Hz), 7.59 (1H, t, *J* = 8.0 Hz), 7.45 (1H, d, *J* = 4.0 Hz), 7.14 (1H, d, *J* = 7.5 Hz), 6.99 (1H, t, *J* = 7.8 Hz), 6.79 (1H, d, *J* = 8.0 Hz), 6.60 (1H, t, *J* = 7.5 Hz), 4.49 (2H, s), 4.76 (2H, t, *J* = 7.0 Hz). ^13^C NMR (DMSO-*d*_6_, 150 MHz, δ, ppm) 159.81, 147.83, 143.36, 141.02, 139.91, 138.41, 137.56, 135.73, 129.90, 129.74, 126.92, 126.77, 124.43, 123.55, 122.54, 121.75, 121.73, 116.25, 116.07, 50.28, 34.84; MS (FAB) *m/z* 435 (MH^+^). Anal. (C_21_H_18_N_6_O_3_S) C, H, N.

### Biology

#### HDAC enzyme assays

The HDAC activity assay was performed using an HDACs/HDAC8 deacetylase fluorometric assay kit (CY-1150/CY-1158, Cyclex Company Limited), HDAC-Glo™ I/II Assay and Screening System (Promega Inc.), HDAC3/HDAC6 fluorescent activity drug discovery kit (AK-531/AK-516, BIOMOL Research Laboratories) or Fluorogenic HDAC Class2α Assay Kit (BPS Bioscience Incorporated) with HDACs (CY-1150, Cyclex Company Limited), HDAC3/NCOR1 complex (SE-515, BIOMOL Research Laboratories), HDAC1 (H83-30G, SignalChem Pharmaceuticals Inc.), HDAC4 (BPS Bioscience Incorporated), HDAC6 (SE-508, BIOMOL Research Laboratories), and HDAC8 (CY-1158, Cyclex Company Limited), according to the supplier’s instructions. The fluorescence of the wells was measured on a fluorometric reader with excitation set at 360 nm and emission detection set at 460 nm, and the values of % inhibition were calculated from the fluorescence readings of inhibited wells relative to those of control wells. The concentration of a compound that results in 50% inhibition was determined by plotting log[Inh] versus the logit function of % inhibition. IC_50_ values were determined by regression analysis of the concentration/inhibition data.

### Western Blot Analysis

HCT116 human colon cancer cells were purchased from American Type Culture Collection (ATCC, Manassas, VA, U.S.A.) and cultured in McCoy’s 5A culture medium containing penicillin and streptomycin, which was supplemented with fetal bovine serum as described in the ATCC instructions. HCT116 cells (1.0×10^5^) were treated for 8 h with 20 µM etoposide and samples at the indicated concentrations in McCoy’s 5A medium, then collected and extracted with SDS buffer. Protein concentrations of the lysates were determined using a Bradford protein assay kit (Bio-Rad Laboratories); equivalent amounts of proteins from each lysate were resolved in AnykD SDS-polyacrylamide gels and then transferred onto nitrocellulose membranes (Bio-Rad Laboratories). After having been blocked for 30 min with Tris-buffered saline (TBS) containing 3% skimmed milk, the transblotted membranes were incubated overnight at 4°C with acetyl NF-κB antibody (CST) (1∶1000 dilution), NF-κB antibody (CST) (1∶1000 dilution), acetyl α-tubulin antibody (Sigma) (1∶2000 dilution), α-tubulin antibody (Sigma) (1∶2000 dilution), acetyl p53 antibody (CST) (1∶500 dilution) or p53 antibody (CALBIOCHEM) (1∶500 dilution) in TBS containing 3% skimmed milk. The membrane was probed with the primary antibody, then washed twice with TBS, incubated with sheep anti-rabbit IgG-horseradish peroxidase conjugates (diluted 1∶1000 for acetyl NF-κB, 1∶2000 for NF-κB or 1∶500 for acetyl p53) or donkey anti-mouse IgG-horseradish peroxidase conjugates (diluted 1∶5000 for acetyl α-tubulin, 1∶5000 for α-tubulin, or 1∶500 for p53) for 1.5 h at room temperature, and again washed twice with TBS and once with TBS-Tween 20 (TBS-T). The immunoblots were visualized by enhanced chemiluminescence.

#### Cell growth inhibition assay

The cells were plated at the initial density of 5,000 cells/well (50 µL/well) in 96-well plates in medium culture and exposed to inhibitors for 48 h in an incubator at 37°C in 5% CO_2_ in air. A solution (5 mg/mL) of 3-(4,5-dimethylthiazol-2-yl)-2,5-diphenyltetrazolium bromide (MTT) was added (10 µL/well) and incubation was continued for 3 h. The solubilized dye was quantified by colorimetric reading at 570 nm. The absorbance values of control wells (*C*) and test wells (*T*) were measured. The absorbance of the test wells (*T*_0_) was also measured at time 0 (addition of compounds). Using these measurements, cell growth inhibition (percentage of growth) by a test inhibitor at each concentration used was calculated as: % growth = 100×[(*T* – *T*_0_)/(*C* – *T*_0_)], when *T*>*T*_0_ and % growth = 100× [(*T* – *T*_0_)/*T*], when *T*<*T*_0_. Computer analysis of the % growth values afforded the 50% growth inhibition parameter (GI_50_). The GI_50_ was calculated as 100× [(*T* – *T*_0_)/(*C* – *T*_0_)] = 50.

#### Viral p24 antigen assay

The p24 antigen level in the cell culture supernatant was measured by p24 antigen capture ELISA assay using a commercial kit (RETRO-TEK HIV-1 p24 Antigen ELISA kit; Zepto Metrix, Buffalo, NY, USA) according to the method reported in ref [54].

#### Molecular modeling

The X-ray structures of HDAC3 and HDAC8 (PDB code 4A69 and 1T64, respectively) were used as the target structures for docking. Protein preparation, receptor grid generation and ligand docking were performed using the Molegro Virtual Docker software package. Compound **T247** was docked into the active site of the protein and was located in a position where the amino group of **T247** can interact with the zinc ion. The standard precision mode of Molegro Virtual Docker was used to determine favorable binding poses, which allowed the ligand conformation to be flexibly explored while holding the protein as a rigid structure during docking.
